# Supplementary material for: Cell-type-specific functionality encoded within the intrinsically disordered regions of OCT4
Source: Nat Commun. 2025 Sep 30;16:8647. doi: 10.1038/s41467-025-63806-3 (PMC12485055; doi:10.1038/s41467-025-63806-3)
Supplement: Supplementary file 1 — Supplementary Information [file 41467_2025_63806_MOESM1_ESM.pdf]

Supplementary Information for the article

Cell-type-specific functionality encoded within the intrinsically disordered regions of OCT4.

Burak Ozkan[1,2†], Mitzy Rios de Anda[1,2†], Elisa Hall-Ponsele[1,2], Maria Rosa Portero Migueles[1], Amani Alshaikh[1,2,3], Marta Hanzevacki[1,2], Moriyah Naama[4], Katharine Furlong[1,2], Gareth A. Roberts[1,2], Meryam Beniazza[1], My Linh Huynh[1], Michael R. O'Dwyer[1,2], Sonia Yiakoumi[1], Christos Spanos[5], Hazar Yassen[4], Keisuke Kaji[1], Hitoshi Niwa[6,7], Yosef Buganim[4], Sally Lowell[1,2], and Abdenour Soufi[1,2*].

[1] Institute of Regeneration and Repair, Centre for Regenerative Medicine, University of Edinburgh, 5 Little France Drive, Edinburgh, EH16 4UU, UK.

[2] Institute of Stem Cell Research, School of Biological Sciences, University of Edinburgh, 5 Little France Drive, Edinburgh, EH16 4UU, UK.

[3] King Abdulaziz City for Science and Technology Health Sector, King Abdullah road, Al Raed District, Riyadh 11442, Saudi Arabia.

[4] Department of Developmental Biology and Cancer Research, Institute for Medical Research Israel-Canada, The Hebrew University-Hadassah Medical School, Jerusalem 91120, Israel.

[5] Wellcome Discovery Research Platform for Hidden Cell Biology, Michael Swann Building, The Kings Buildings Campus, Edinburgh, EH9 3BF.

[6] Institute of Molecular Embryology and Genetics, Kumamoto University, 2-2-1 Honjo, Chuo-ku, Kumamoto, 860-0811, Japan.

[7] RIKEN Center for Developmental Biology, 2-2-3 Minatojima-minamimachi, Chuo-ku, Kobe 6500047, Japan.

[†] Authors contributed equally to this work

[*] Corresponding Author: Abdenour.Soufi@ed.ac.uk

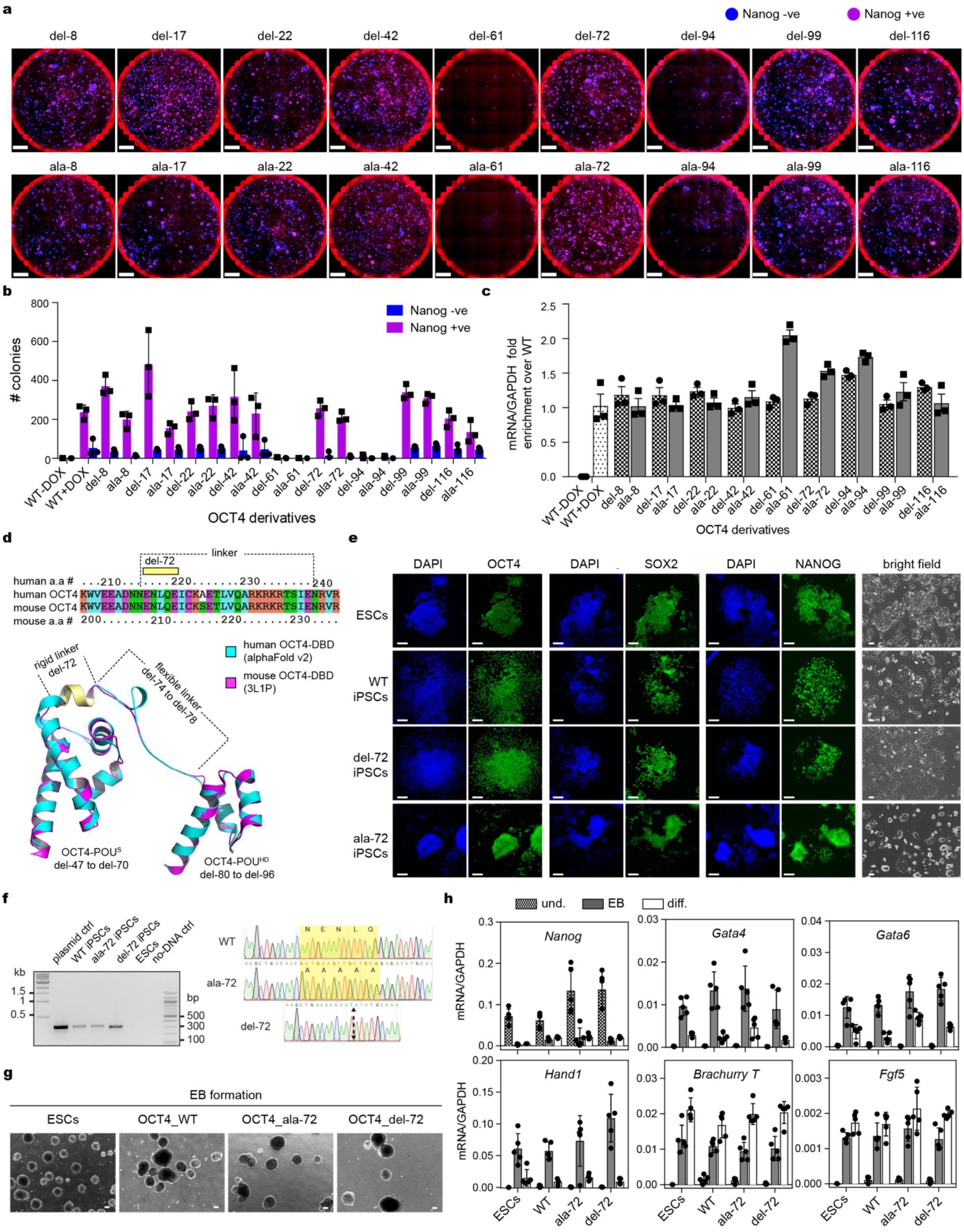

Supplementary Fig. 1: OCT4 with alanine-stretch substitution or the equivalent deletion show similar reprogramming activity to iPSCs.

a, Representative whole-well images of merged (magenta) DAPI fluorescence (blue) and Nanog immunofluorescence (red) on day 16 of reprogramming using selected deletions (del.) and alanine-stretch substitution mutants (ala.) of OCT4. Scalebar, 5mm. **b,** Bar plot showing the efficiency of reprogramming as shown in **(a)**, by counting Nanog positive iPSC colonies (magenta bars) versus DAPI alone colonies (blue bars). OSKM -/+ Dox were used as controls. **c,** The expression of selected OCT4 del. (black) and ala. (grey) mutants as measured by qRT–PCR in OSKM-48h. **d,** 3D homology alignment of human (cyan, alphaFold2) and mouse (magenta, PDB:3L1P) OCT4-DBD, showing the position of del-72 (yellow) within the rigid linker region. Sequence alignment of the linker is shown on the top. **e,** Immunofluorescence images showing Oct4, Sox2, and Nanog (green) expression in iPSCs generated using OCT4 WT, ala-72 and del-72 mutants, compared to ESCs. DAPI staining (blue) and bright field images are also shown. Scalebar, 100µm. **f,** Genotyping iPSC lines by PCR and Sanger sequencing confirming the presence of the expected deletion or alanine substitution (yellow) when using OCT4 del-72 and ala-72 mutants to generate iPSCs, respectively. These mutations were absent in iPSCs generated using OCT4 WT. Uncropped images are provided in Supplementary Fig. 10. **g,** Embryoid bodies (EBs) generated from iPSCs using OCT4 WT, ala-72 and del-72 mutants. **h,** EBs from panel **(g)** can differentiate spontaneously *in vitro* (diff.) into all three germ layers, as assessed by RT-qPCR of relevant markers as indicated. Undifferentiated iPSCs (und.) were used as controls. Data are presented as mean values +/- SEM from n=3 (**b** and **c**) and n=5 **(h)** independent biological replicates. Images are representatives of n=3 biological replicates (**a, e** and **g**).

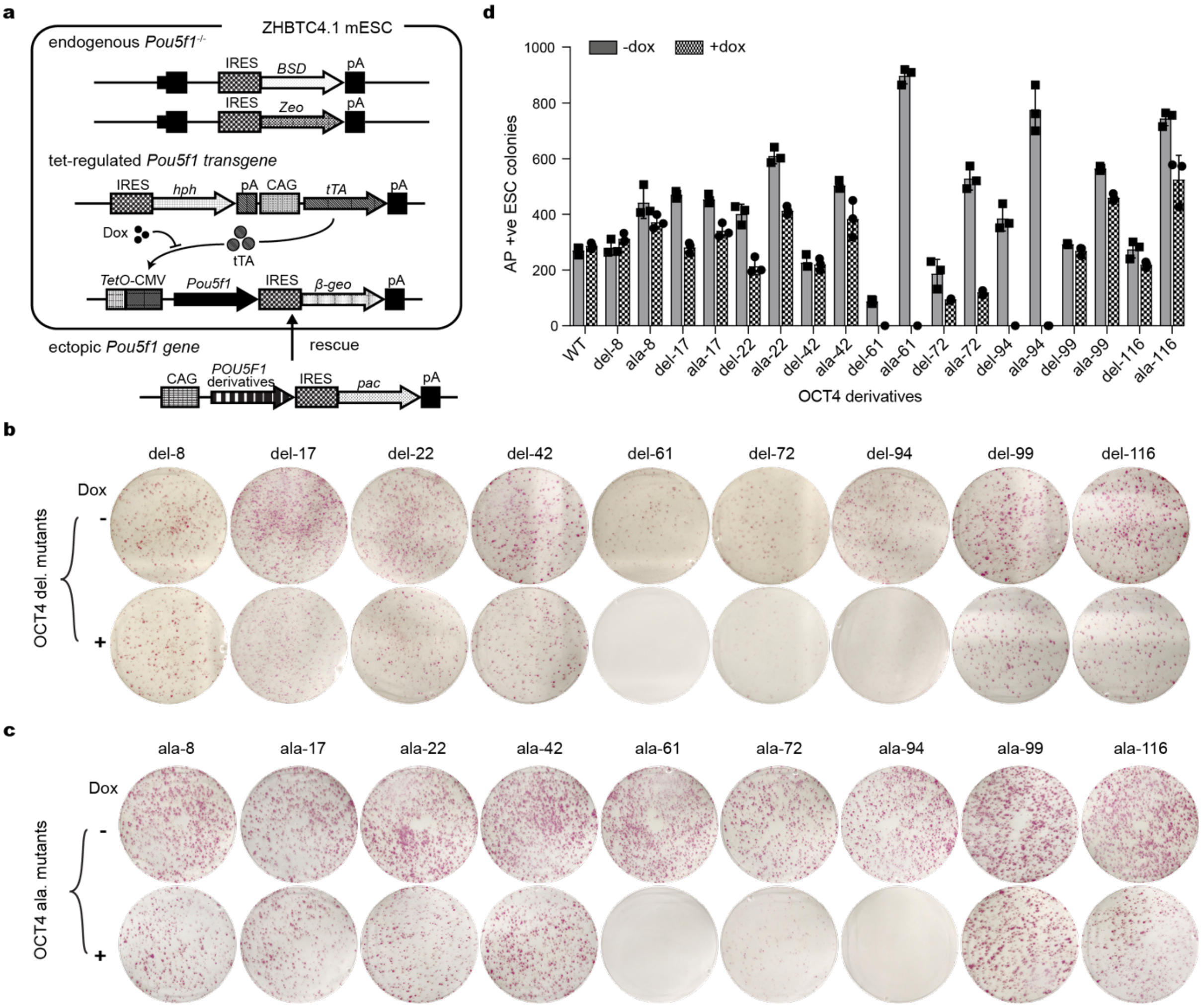

Supplementary Fig. 2: OCT4 deletion mutants and the equivalent alanine-stretch substitutions show similar ESC self-renewal.

a, A schematic showing the strategy for downregulating the *Pou5f1* transgene in ZHBTc4.1 mESCs using Dox. The ZHTc4.1 mESCs have endogenous *Pou5f1* alleles knocked out. The self-renewal of ZHTc4.1 mESCs can be rescued in the presence of Dox when transfected with an ectopic *Pou5f1* gene**. b,c,** Representative whole-well images showing AP staining of ZHBTC4.1 ESC colonies rescued by the ectopic expression of *Pou5f1* del. mutants (b) and ala. mutants **(c)** after treatment with Dox. Images are representative of n=3 independent biological replicates. **d,** Bar plot showing the efficiency of pluripotency rescue, which was quantified by counting the number of AP positive colonies as shown in (b,c) in the presence and absence of Dox.

Data are represented as mean values +/- SEM of n=3 independent biological replicates.

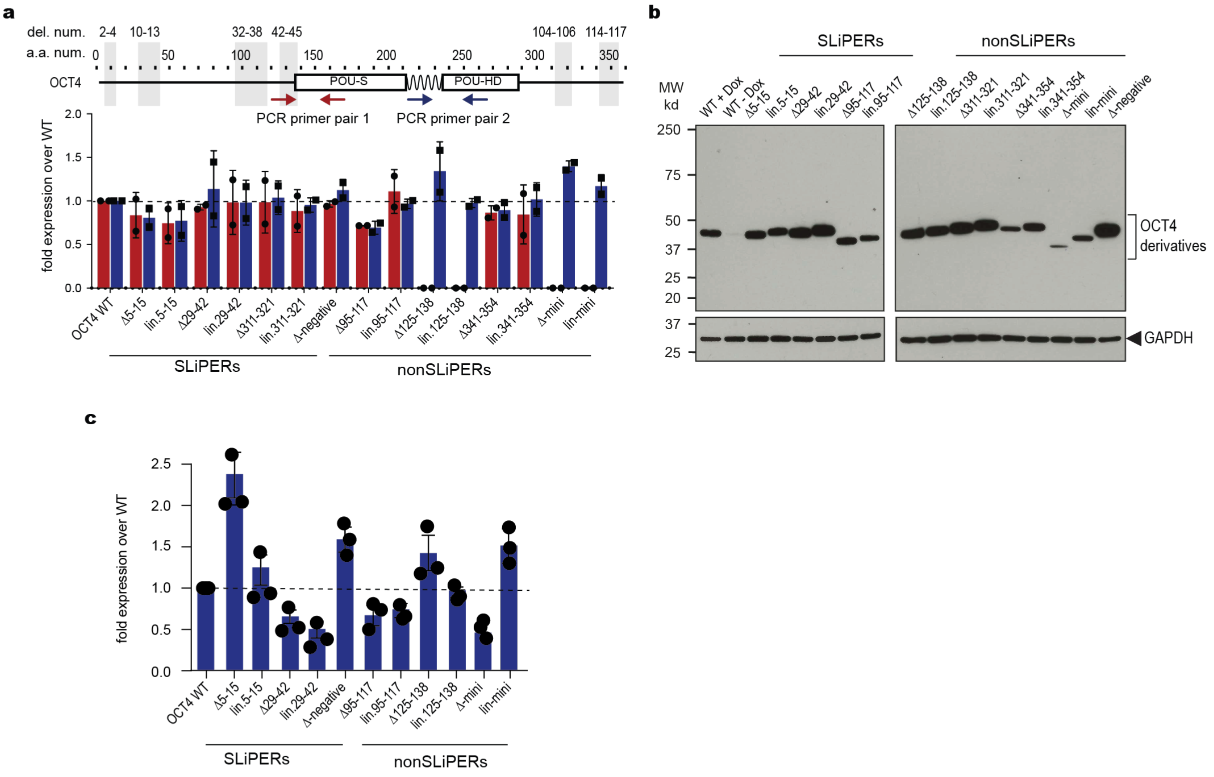

Supplementary Fig. 3: OCT4 SLiPER and non-SLiPER mutants show similar expression during early reprogramming.

a, Bar plot showing the expression of OCT4 truncated mutants relative to WT (dotted line) as measured by qRT–PCR in OSKM-48h using two pairs of primers (red and blue bars). A schematic of OCT4 defined domains (grey) with the red and blue arrows representing the primer pairs used for qPCR. **b,** Protein levels of OCT4 WT and truncated mutants measured by Western blot analysis. GAPDH levels were used as loading control and all proteins migrated according to their expected sizes as indicated. Uncropped images are provided in Supplementary Fig. 11. **c,** Bar plot showing the expression of OCT4 truncated mutants relative to WT as measured by qRT–PCR in ZHBTc4.1 mESCs during pluripotency rescue assay using primer pair 2 shown in **(a)**. Data are presented as mean values +/- SEM of n=2 **(a)** and n=3 **(c)** biological replicates relative to WT (dotted line).

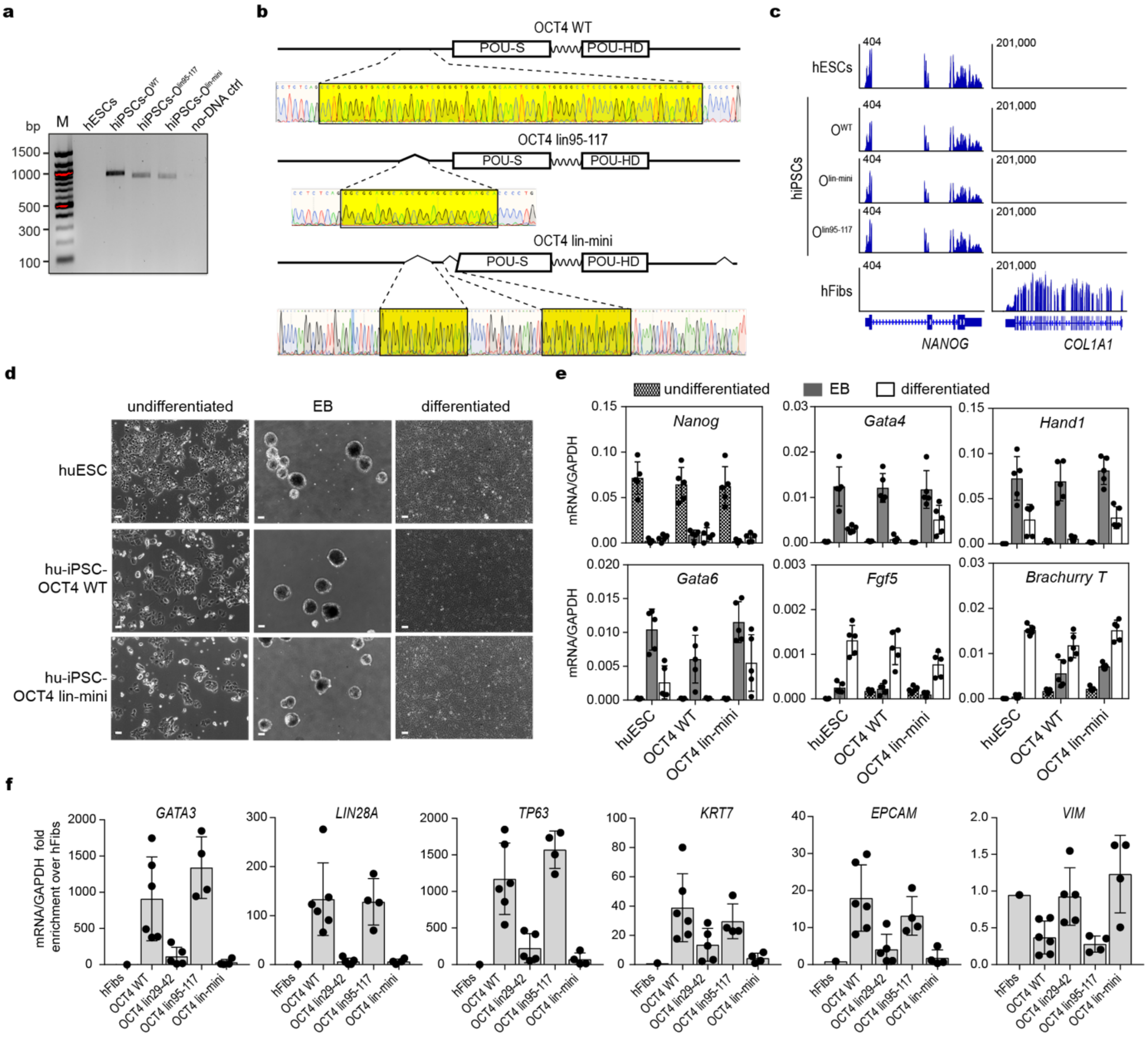

Supplementary Fig. 4: Reprogramming to human pluripotent and trophoblast stem cells can be dissected apart using different OCT4 domains.

a, b, Genotyping of iPSC lines by PCR **(a)** and Sanger sequencing **(b)** of the lentiviral-integrated OCT4 coding sequence to confirm the presence of the expected linker insertion (yellow) when using OCT4 lin-95-117 mutant to generate iPSCs from human fibroblasts. These mutations were absent in control iPSCs lines generated using OCT4 WT. Uncropped images are provided in Supplementary Fig. 12. **c,** Screen shots of genome browser tracks showing the expression levels of the pluripotency marker *NANOG* (left) and the fibroblast marker *COL1A1* (right) in human ESCs, iPSCs and

fibroblasts. **d,** Embryoid bodies (EB) generated from iPSC lines using OCT4 WT and lin-mini mutant as compared to mouse ES cells. Scalebar, 100µm. **e,** EBs from panel **(d)** can differentiate spontaneously *in vitro* (diff.) into all three germ layers, as assessed by RT-qPCR of relevant markers as indicated. Undifferentiated iPSCs (und.) were used as controls. **f,** The expression of selected TSC markers (*GATA3, LIN28A, TP63, KRT7*, and *EPCAM*) and the fibroblast marker (*VIM*) mRNA measured by qRT–PCR and normalized against *GAPDH* after reprogramming to iTSCs. Data are represented as mean values +/- SEM of n=5 **(e)** and n=6 **(f)** biological replicates. Images are representatives of n=3 biological replicates **(d)**.

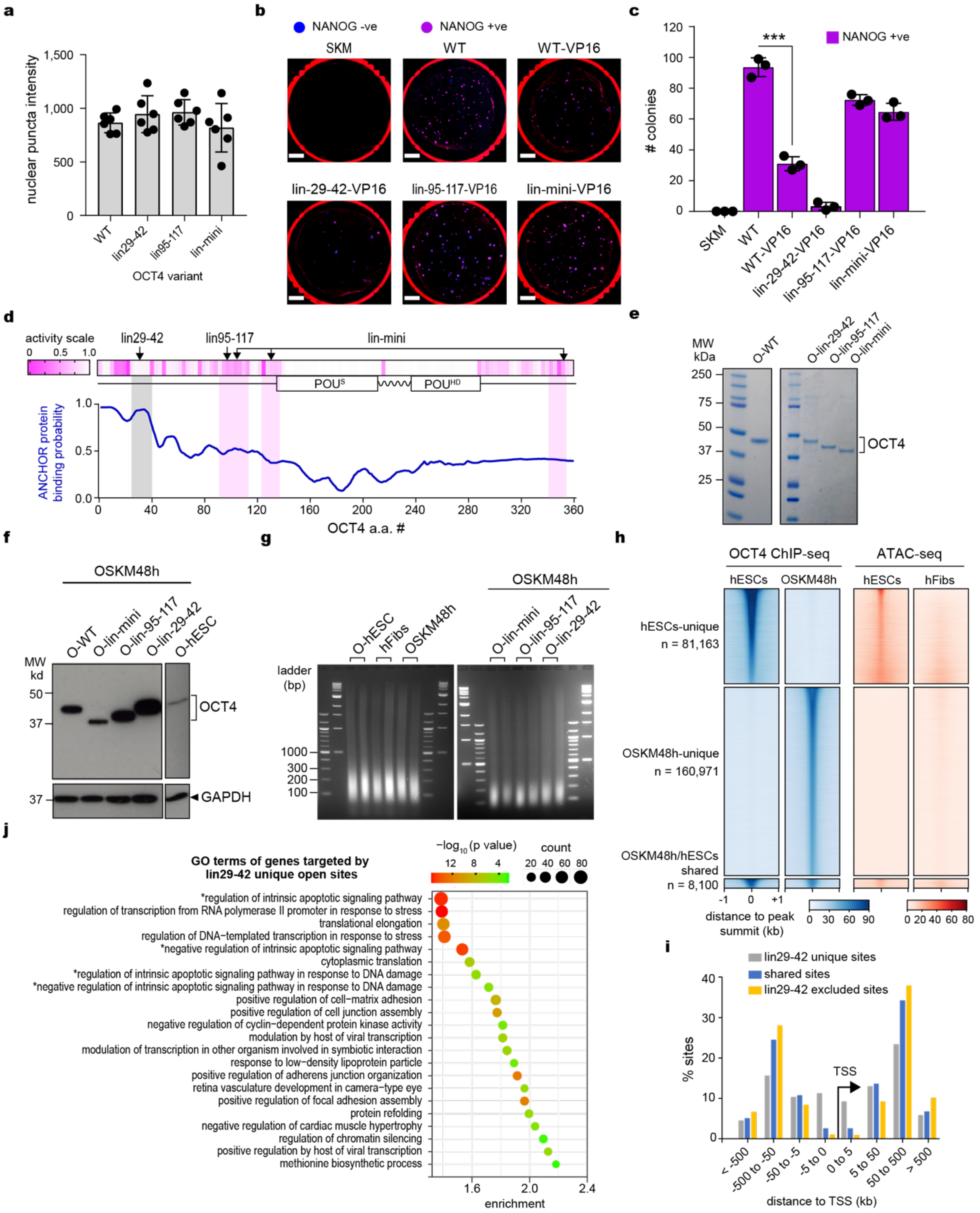

Supplementary Fig. 5: Removing SLiPERs change OCT4 engagement with the somatic genome.

a, Quantification of OCT4 foci shown in Fig 3b. **b,** Representative whole-well images of merged (magenta) DAPI fluorescence (blue) and Nanog immunofluorescence (red)

after reprogramming hFibs to iPSCs using various VP16AD-OCT4 fusion derivatives. Scalebar, 5mm. **c,** Bar plot showing Nanog positive iPSC colonies (magenta bars) generated by VP16AD-OCT4 fusion derivatives compared to OCT4 wildtype. SKM was used as control. **d,** Graph of protein-protein interaction probability within OCT4 as calculated by the ANCHOR algorithm (blue). Heatmap of OCT4 reprogramming activity and a schematic of OCT4-structural domains are indicated above. SLiPERs and nonSLiPERs are highlighted in red and green, respectively. **e,** Purified recombinant OCT4 WT and mutants analysed by SDS-PAGE and Coomassie staining. The respective OCT4 bands run at the expected size when compared to the sizes of the protein standards (M lanes). **f,** Protein levels of OCT4 WT and mutants in OSKM-48h and hESCs. GAPDH levels were used as loading control and all proteins migrated according to their expected sizes as indicated. **g,** Agarose gel of sonicated chromatin from OSKM-48h, fibroblasts, and hESCs. **h,** ChIP-seq read density heatmaps (blue) of OCT4 in OSKM-48h and human ESCs relative to ATAC-seq (red) spanning ± 1kb from OCT4 peak summits. The sequences were rank-ordered according to ChIP-seq read density. The number of sites (n) of each group and the enrichment colour scale (RPGC) are indicated. **i,** GREAT (Genomic Regions Enrichment of Annotations Tool) analysis showing the distance to TSS distribution of ChIP-seq peaks enriched uniquely or shared between OCT4 WT and Lin-29-42 mutant during early reprogramming. **j,** GO terms identified by GREAT for genes targeted uniquely by OCT4 lin-29-42 (reprogramming deficient) mutant in early reprogramming. Data are represented as mean values +/- SEM of n=6 **(a)** and n=3 **(c)** biological replicates. Images are representatives of n=3 biological replicates (**b** and **f**). Uncropped images are provided in Supplementary Fig. 13-15.

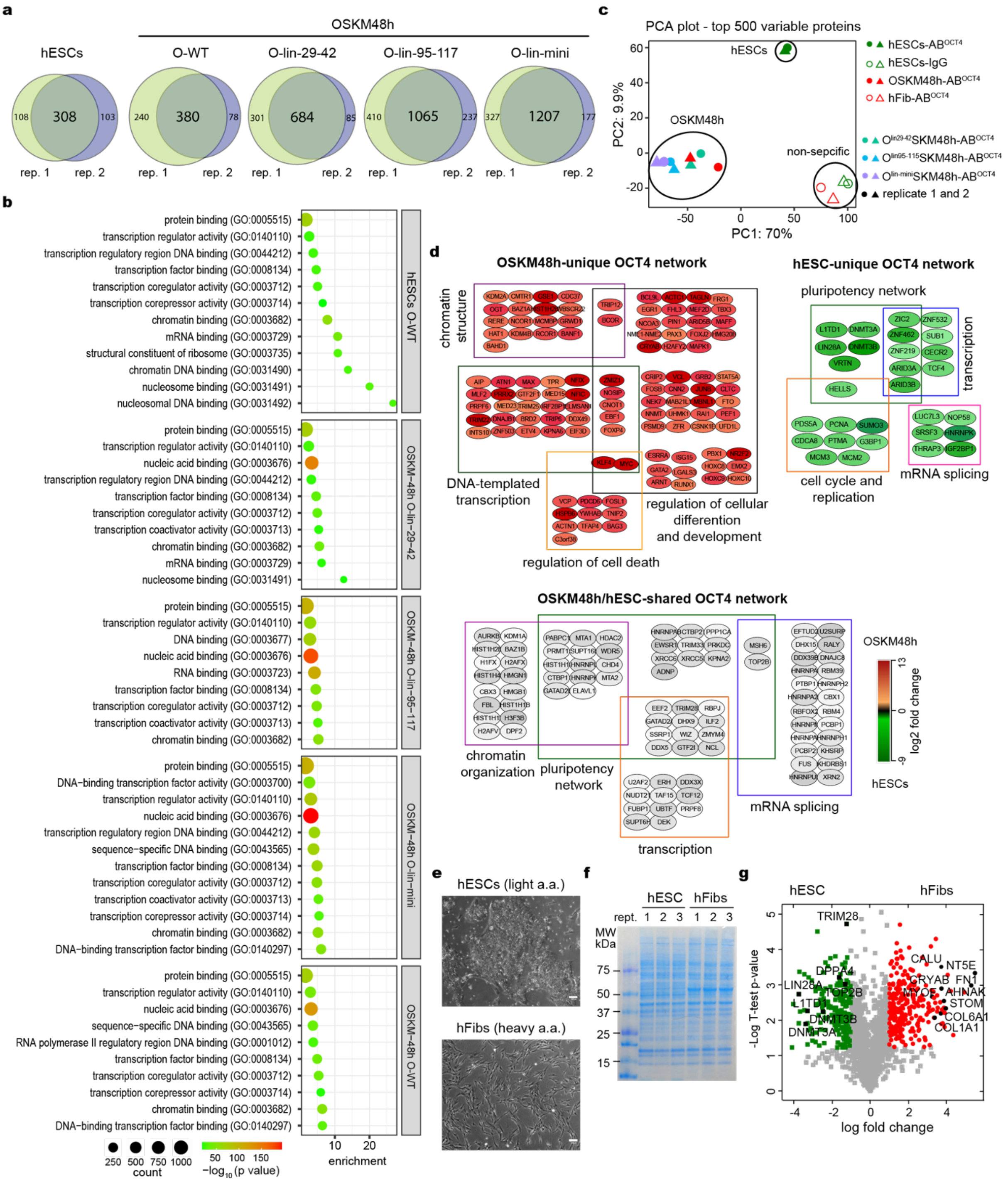

Supplementary Fig. 6: OCT4 engagement of the somatic proteome is markedly different from that in ESCs.

a, Venn diagrams illustrating the significant overlap between OCT4-associated proteins from two biological replicates (n=2) as identified by ChIP-SICAP for OCT4 WT and mutants in OSKM-48h and hESCs. **b,** GO terms for proteins associated with

OCT4 in both replicates shown in **(a)**. **c,** PCA plot of differentially enriched proteins identified by ChIP-SICAP on OCT4 WT and mutants in OSKM-48h and hESCs compared to controls. **d,** STRING network analysis of OCT4-interacting partners uniquely enriched in OSKM-48h (red), in ESCs (green) or equally enriched in both (grey) grouped by their functional association (rectangles). The proteins are colour coded according to the log2 fold change colour scale shown on the side. **e,** Bright field images of ESCs and hFibs grown in light and heavy a.a. SILAC-compatible media for 5 passages, respectively. Images are representatives of n=3 biological replicates. Scalebar, 100µm. **f,** Light and heavy a.a. labelled proteins extracted from ESCs and hFibs shown in **(e)** and run on SDS-PAGE before trypsin digest and MS. Uncropped image shown supplementary fig. 16. **g,** Volcano plot showing the differential abundance of proteins in hFibs and ESCs as identified by SILAC MS. The −log10 unpaired t-test FDR-adjusted P value is plotted against the log2 (fold change). Log2 (fold change) > 1.5 with FDR < 0.1 are considered significantly more enriched in ESCs (green) over hFibs, or vice versa (red). Example proteins from the unique categories are indicated.

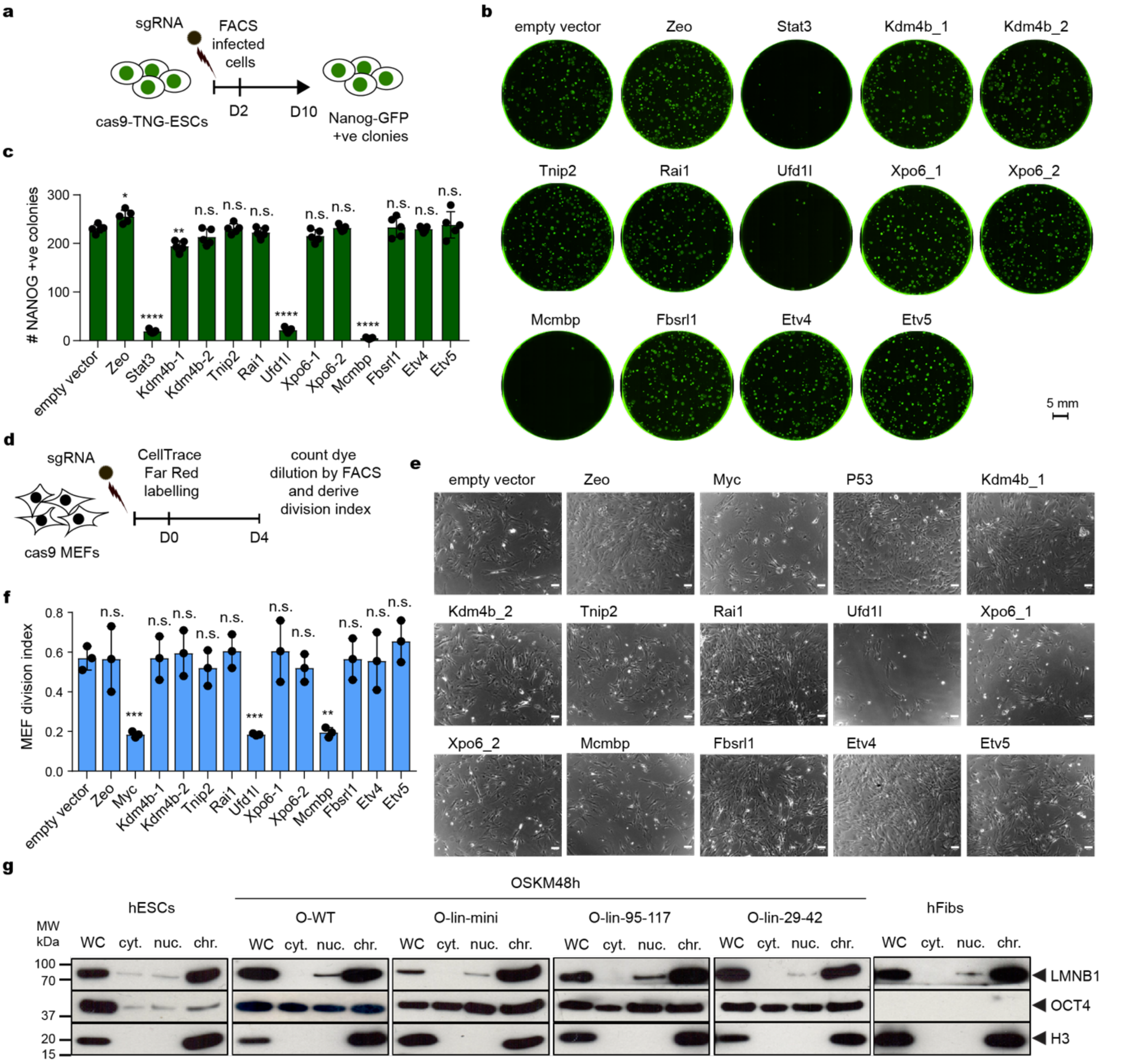

Supplementary Fig. 7: OCT4 recruits unique set of proteins to induce pluripotency.

a, Experimental flow chart of clonogenicity assay to test the effect of candidate gene (indicated above) KO on ESC self-renewal. **b,** Whole well images showing Nanog +ve ESC colonies 10 days after KO of gene candidates. Scalebar, 5mm. **c,** Bar plots of Nanog +ve ESC colonies in **(b)**. **d,** Experimental flowchart to test the effects of gene candidates KO on the proliferation of cas9-MEFs using CellTrace^{TM}. **e,** Bright field images showing the effects of gene candidate KO on MEFs after 4 days in culture. Myc KO, which blocks proliferation and P53 KO, which enhances proliferation, were

used as controls. Scalebar, 100µm. **f,** Bar plot showing the effect of candidate gene KO on the proliferation of MEFs shown in (e) by extracting the division index from CellTrace labelling dilution as quantified by FACS. Due to the high proliferation rate of P53 KO cells, which resulted in highly diluted CellTrace far red dye, accurate division index could not be derived. **g,** Western blot analysis showing the cellular fractionation of hFibs, OSKM48h (using OCT4 WT and mutants), and hESCs. Whole cell extracts (WC) were used as control. The nuclear envelope protein LMNB1 was used as a marker for the nuclear fractions (nuc.) and should not be present in the cytoplasmic (cyt.) fractions. The histone H3 was used as a marker of chromatin (chr.) fraction. Data presented as mean values +/- SEM of n=3 independent biological replicates (**c** and **f**). Statistical significance was assessed by one-way ANOVA multiple comparison and indicated by adjusted P values (n.s. > 0.05, (**P < 0.01, ***P < 0.001, and ****P < 0.0001) (**c** and **f**). Images are representative of n=3 biological replicates (**b, e** and **g**). uncropped images are shown in supplementary fig. 17.

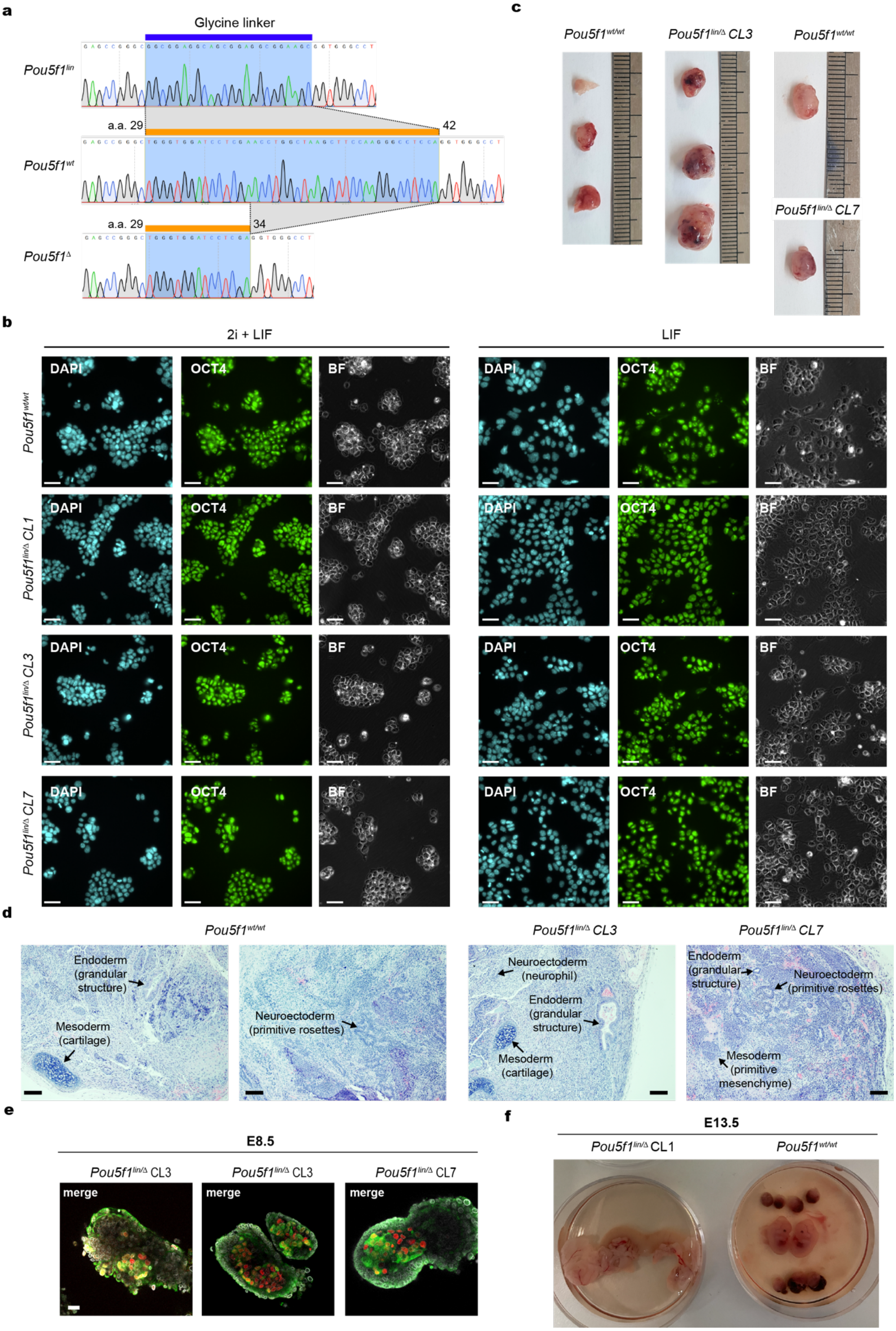
a
Glycine linker
Pou5f1lin
a.a. 29
42
Pou5f1wt
a.a. 29
34
Pou5f1Δ
b
2i + LIF
LIF
Pou5f1wt/wt
Pou5f1lin/Δ CL1
Pou5f1lin/Δ CL3
Pou5f1lin/Δ CL7
DAPI
OCT4
BF
c
Pou5f1wt/wt
Pou5f1lin/Δ CL3
Pou5f1wt/wt
Pou5f1lin/Δ CL7
d
Pou5f1wt/wt
Pou5f1lin/Δ CL3
Pou5f1lin/Δ CL7
Endoderm (grandular structure)
Mesoderm (cartilage)
Neuroectoderm (primitive rosettes)
Neuroectoderm (neurophil)
Endoderm (grandular structure)
Mesoderm (cartilage)
Endoderm (grandular structure)
Neuroectoderm (primitive rosettes)
Mesoderm (primitive mesenchyme)
e
E8.5
Pou5f1lin/Δ CL3
Pou5f1lin/Δ CL3
Pou5f1lin/Δ CL7
merge
f
E13.5
Pou5f1lin/Δ CL1
Pou5f1wt/wt

Supplementary Fig. 8: Removing OCT4 SLiPERs blocks embryos developing beyond late gastrulation.

a, Genotyping of mouse ESC lines targeted by CRISPR-cas9 for linker knock-in. Sanger sequencing confirms the presence of the expected linker substitution in one allele and part-deletion of the same region on the other allele (shaded in blue). **b,** Representative immunofluorescence images showing similar expression of OCT4 (green) in *Pou5f1*$^{wt/wt}$ and *Pou5f1*$^{lin/Δ}$ ES lines under LIF/2i and LIF culture conditions. DAPI staining (blue) and bright field images are also shown. Images are representatives of n=9 replicates. Scalebar, 100µm. **c,** Teratomas generated from *Pou5f1*$^{wt/wt}$ and *Pou5f1*$^{lin/Δ}$ mutant ESC lines to indicate their pluripotency. Uncropped images are shown in supplementary figure 18. **d,** Histology of teratomas derived from ESCs *Pou5f1*$^{wt/wt}$ and *Pou5f1*$^{lin/Δ}$ mutant lines shown in **(c)**, showing the presence of various tissues originated from all three germ lines. Scalebar, 100µm. **e,** Immunofluorescence images of LaminB1 (grey), Nanog (red), and GFP (green), showing the contribution of *Pou5f1*$^{lin/Δ}$ CL3 and CL7 ESC lines to E8.5 embryos. Scalebar, 20µm. **f,** Example of chimeric embryos and foetal resorption generated from ESCs *Pou5f1*$^{wt/wt}$ and *Pou5f1*$^{lin/Δ}$ CL1 line by mid-gestation E13.5.

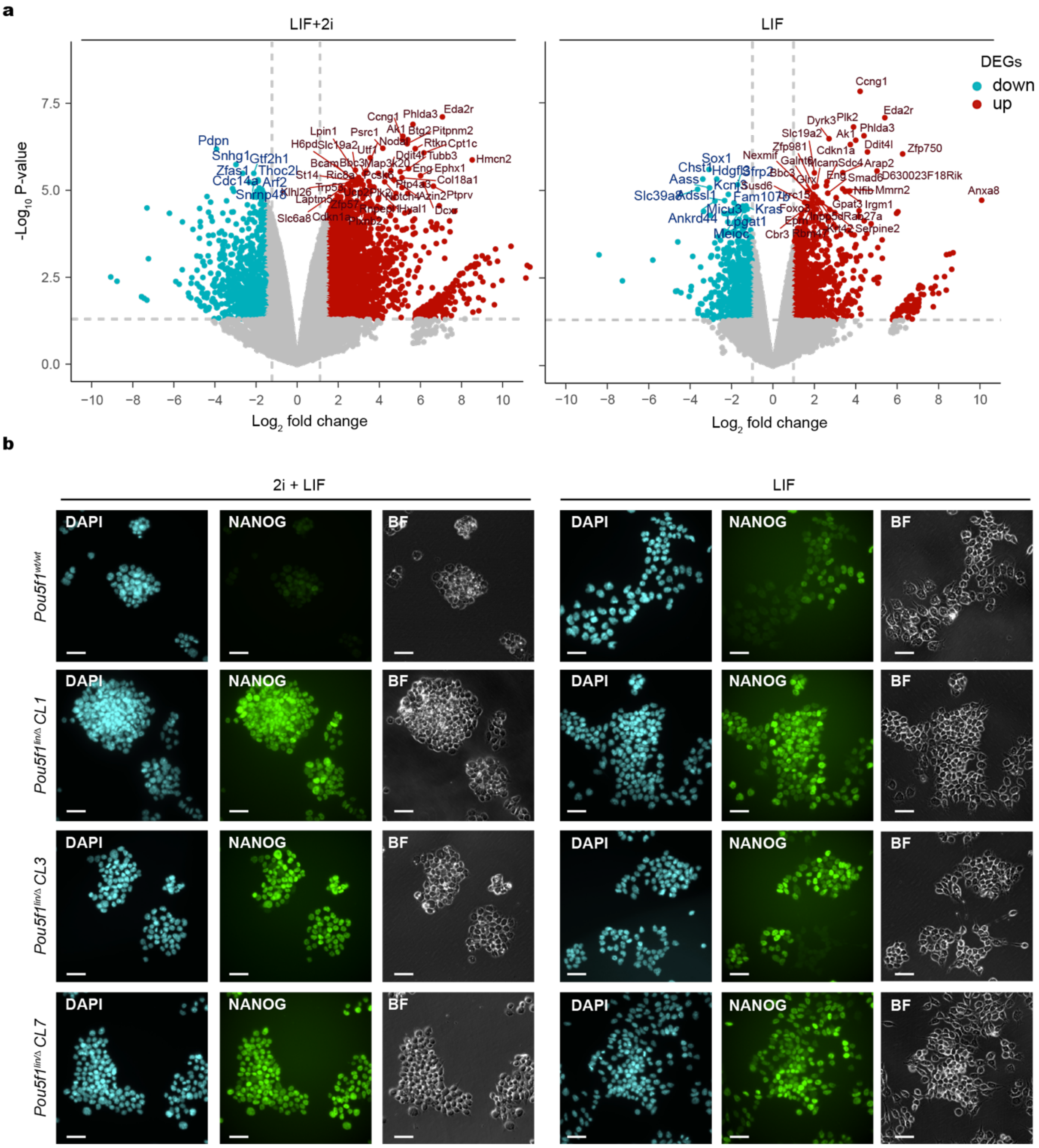

Supplementary Fig. 9: Removing OCT4 SLiPERs blocks ESCs from exiting pluripotency.

a, Volcano plots showing the differentially expressed genes between *Pou5f1*$^{lin/\Delta}$ and *Pou5f1*$^{wt/wt}$ ESC lines under LIF+2i and LIF culture conditions. Examples of

upregulated and downregulated genes are indicated. The significance threshold is represented by dotted lines denoting log2 (fold change) of 1.5 and FDR adjusted P value of 0.05 by unpaired t-test. **b,** Representative immunofluorescence images showing elevated expression of Nanog (green) in *Pou5f1*$^{lin/\Delta}$ mutant lines compared to *Pou5f1*$^{wt/wt}$ ESCs under LIF+2i and LIF culture conditions. DAPI staining (blue) and bright field (BF) images are also shown. Scalebar, 100µm. Images are representative of n=9 replicates.

Source data for Supplementary Information

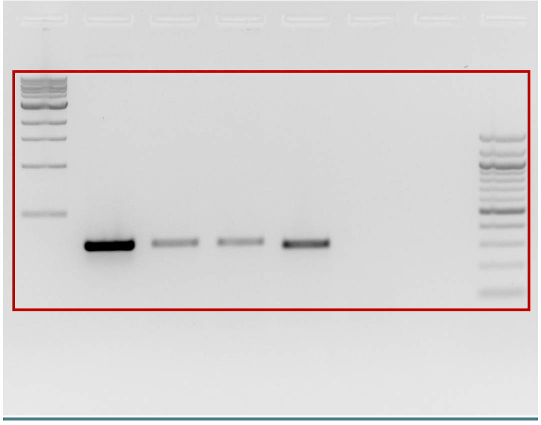

Supplementary Fig. 10: uncropped agarose gel image of iPSC genotyping PCR shown in supplementary fig. 1f.

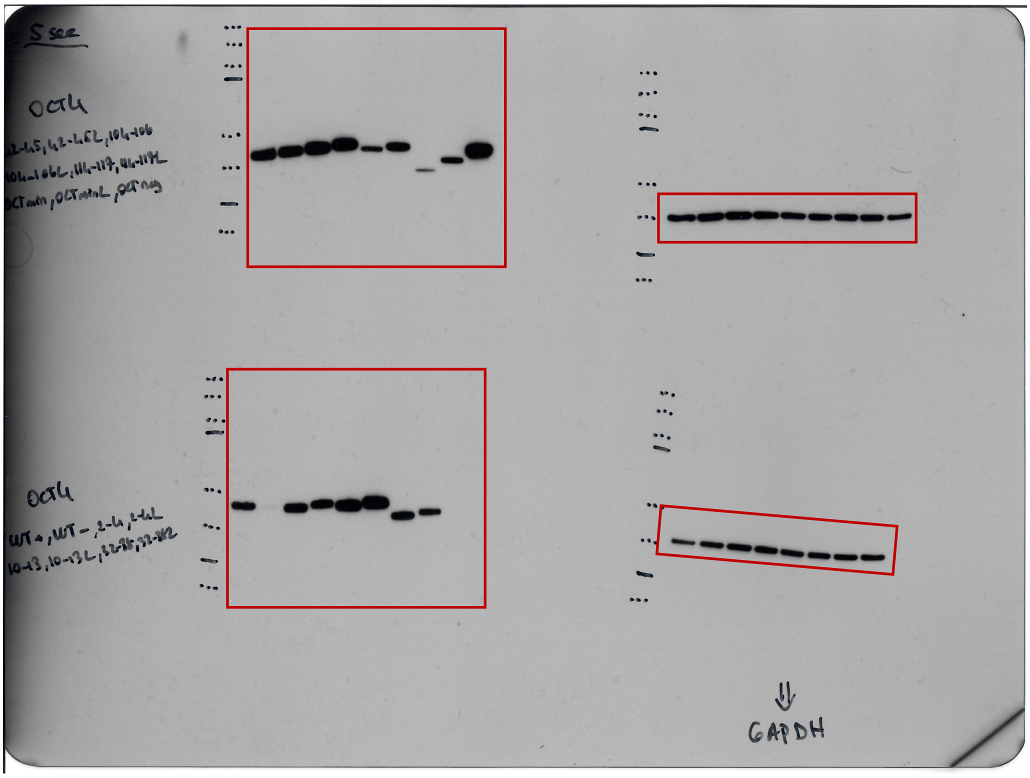

Supplementary Fig. 11: uncropped western blots of OCT4 variant expression shown in supplementary fig. 3b.

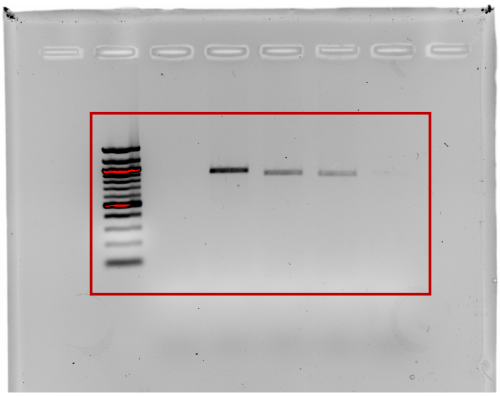

Supplementary Fig. 12: uncropped agarose gel image of human iPSC genotyping PCR shown in supplementary fig. 4a.

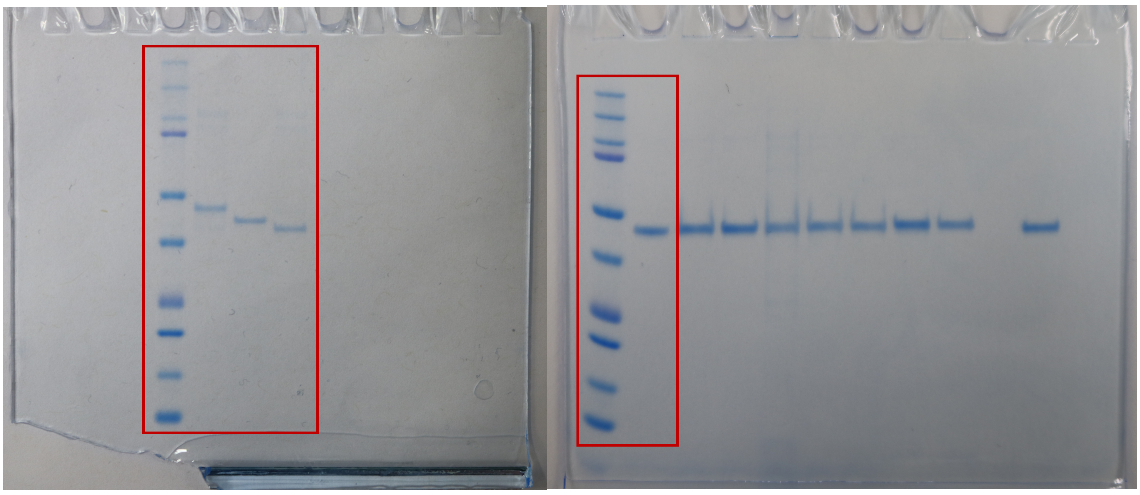

Supplementary Fig. 13: uncropped SDS-PAGE of recombinant OCT4 variants shown in supplementary fig. 5e.

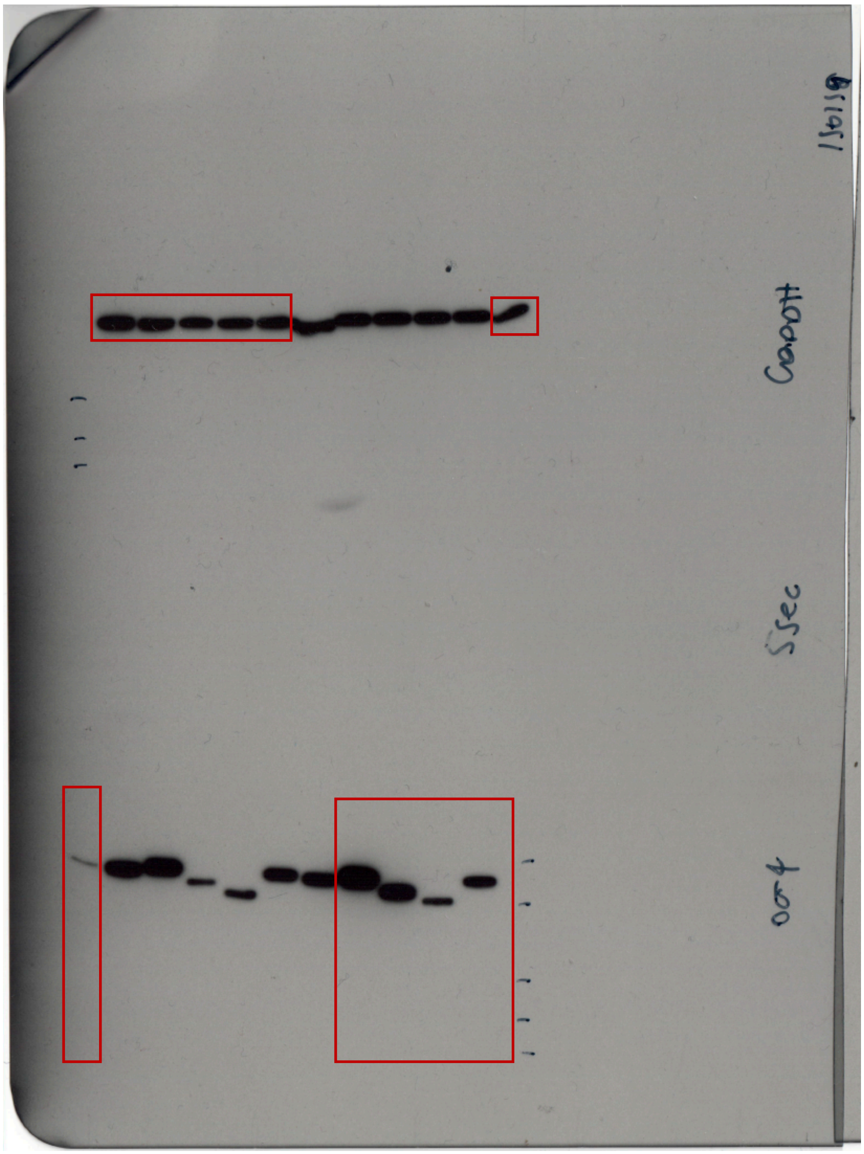

Supplementary Fig. 14: uncropped Western blots of OCT4 variants shown in supplementary fig. 5f.

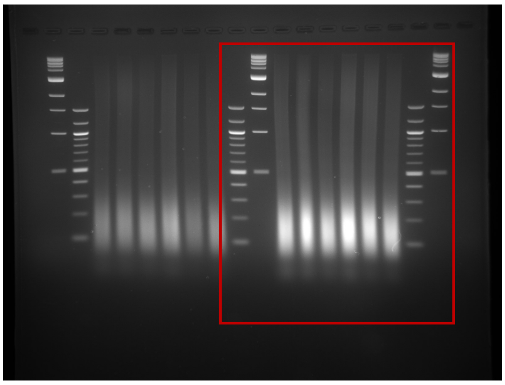

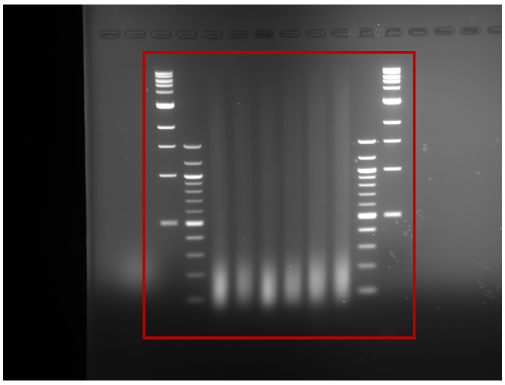

Supplementary Fig. 15: uncropped agarose gels of chromatin sonication shown in supplementary fig. 5g.

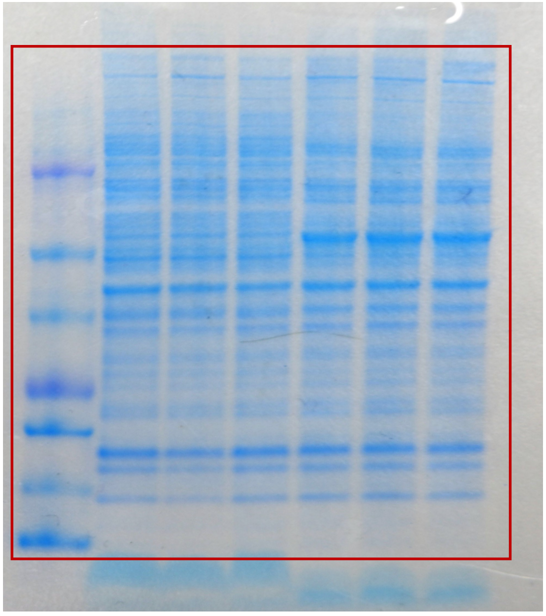

Supplementary Fig. 16: uncropped SDS-PAGE of proteins extracted for SILAC-MS shown in supplementary fig. 6f.

Anti-LaminB

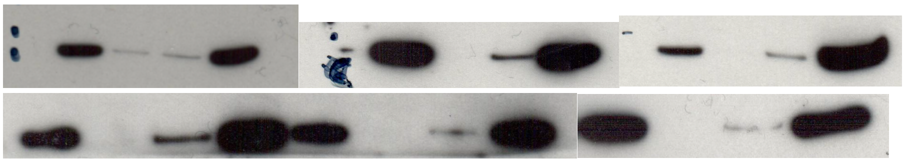

Anti-OCT4

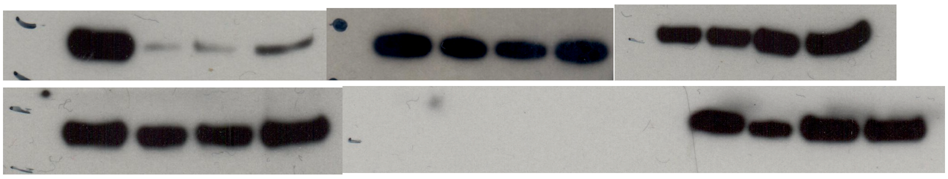

Anti-H3

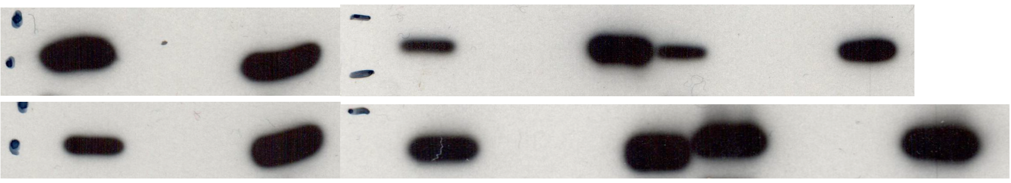

Supplementary Fig. 17: uncropped Western blots of cellular fractionation shown in Supplementary Fig. 7g.

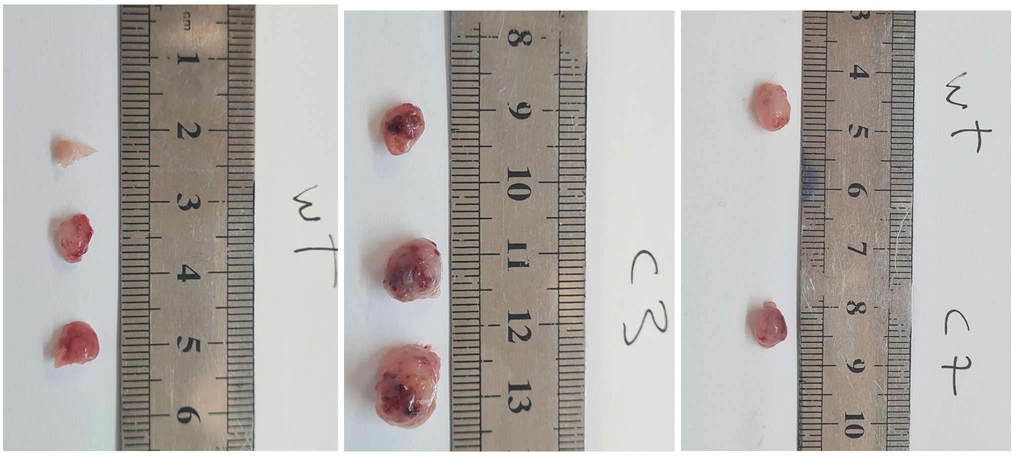

Supplementary Fig. 18: uncropped photos of teratomas shown in supplementary fig. 8c.
