# Supplementary material for: Cell-type-specific functionality encoded within the intrinsically disordered regions of OCT4
Source: Nat Commun. 2025 Sep 30;16:8647. doi: 10.1038/s41467-025-63806-3 (PMC12485055; doi:10.1038/s41467-025-63806-3)
Supplement: Supplementary file 3 — Description of Additional Supplementary Files [file 41467_2025_63806_MOESM3_ESM.pdf]

File Name: Supplementary Data 1

Description: DNA sequences of OCT4 mutants used in this study, showing the size in bp and expected molecular weight of the protein in KDa.

File Name: Supplementary Data 2

Description: PCR Primer sequences used for molecular cloning of OCT4 mutants into the different expression vectors.

File Name: Supplementary Data 3

Description: The complementary DNA Oligos used for cloning sgRNA into the lentivirus vector. The sgRNA target genes are indicate.

File Name: Supplementary Data 4

Description: DNA sequences of primers used for qPCR. Gene names for the primers are indicated with comments on detection.

File Name: Supplementary Data 5

Description: Raw data detected by MS in OCT4 ChIP-SICAP experiments.

File Name: Supplementary Data 6

Description: Normalized data of MS used for differential analysis of OCT4 ChIP-SICAP. The relative amount of proteins in two biological samples were expressed as label-free quantification (LFQ) intensity and fold change and significance are indicated.
