# Supplementary material for: Cell-type-specific functionality encoded within the intrinsically disordered regions of OCT4
Source: Nat Commun. 2025 Sep 30;16:8647. doi: 10.1038/s41467-025-63806-3 (PMC12485055; doi:10.1038/s41467-025-63806-3)
Supplement: Supplementary file 5 — Reporting Summary [file 41467_2025_63806_MOESM5_ESM.pdf]

Corresponding author(s): Abdenour

Last updated by author(s): Aug 22, 2025

Reporting Summary

Nature Portfolio wishes to improve the reproducibility of the work that we publish. This form provides structure for consistency and transparency in reporting. For further information on Nature Portfolio policies, see our Editorial Policies and the Editorial Policy Checklist.

Statistics

For all statistical analyses, confirm that the following items are present in the figure legend, table legend, main text, or Methods section.

n/a	Confirmed	
☐	☒	The exact sample size (n) for each experimental group/condition, given as a discrete number and unit of measurement
☐	☒	A statement on whether measurements were taken from distinct samples or whether the same sample was measured repeatedly
☐	☒	The statistical test(s) used AND whether they are one- or two-sided<br>*Only common tests should be described solely by name; describe more complex techniques in the Methods section.*
☐	☒	A description of all covariates tested
☐	☒	A description of any assumptions or corrections, such as tests of normality and adjustment for multiple comparisons
☐	☒	A full description of the statistical parameters including central tendency (e.g. means) or other basic estimates (e.g. regression coefficient) AND variation (e.g. standard deviation) or associated estimates of uncertainty (e.g. confidence intervals)
☐	☒	For null hypothesis testing, the test statistic (e.g. F, t, r) with confidence intervals, effect sizes, degrees of freedom and P value noted<br>*Give P values as exact values whenever suitable.*
☒	☐	For Bayesian analysis, information on the choice of priors and Markov chain Monte Carlo settings
☒	☐	For hierarchical and complex designs, identification of the appropriate level for tests and full reporting of outcomes
☐	☒	Estimates of effect sizes (e.g. Cohen's d, Pearson's r), indicating how they were calculated

Our web collection on statistics for biologists contains articles on many of the points above.

Software and code

Policy information about availability of computer code

Data collection	Sequencing data was collected using the Illumina 2500, 4000 and NovaSeq platforms.<br>Western blots were imaged using BioRAD ChemiDoc MP imaging system.<br>fluorescent immunostaining images were captured by Nikon Eclipse T! microscope, Leica SP8 confocal microscope and IRIS Digital Cell Imaging System. Whole well images were taken using CELIGO image cytometer<br>DNA fragment sizes were measured by Agilent 2200 Tapestation and DNA concentration was measured by Qubit Fluorometric Quantitation.<br>Mass spec data were collected using the Thermo Scientific™ Orbitrap Fusion Lumos™ Tribrid™ mass spectrometer.FACS<br>FACS data acquisition using LSRFortessa (BD Biosciences) cytometer.<br>Luciferase data was collected using Promega Glomax Multi Detection System.

Data analysis	Next generation data was analyzed using the following softwares: FASTQC v0.11.9, Bowtie 2.3.4.1, Bedtools v2.18, Picard v2.20, MACS v2.1.1, DeepTools v3.5.4, Qualimap v2.2.1, SAMTool2 v1.3.1, BEDOPS V2.49, MEME v5.0.2, R v3.6 (various R packages as indicated in the methods), Cutadapt v3.3, STAR v2.5.3a, Subread v1.5.2, DESeq2.<br>Mass spec data was analyzed using: Cytoscape v3.8, STRING app v2.2, DyNet Analyser app v1.0.0.<br>protein structure and prediction was analyzed using: AlphFold2, PONDR2, ANCHOR2 and PyMOL Molecular Graphics v 3.0.3.<br>Plots were generated using Garphpad Prism 7 and various other packages in R v3.4 including ggplot2.<br>FACS data was analyzed using Flowjo v10.<br>Fluorescent microscope images were analyzed using ImageJ2.<br>SDS-PAGE and EMSA images were analyzed using FujiFim Multi Gauge image software ver2.0.

For manuscripts utilizing custom algorithms or software that are central to the research but not yet described in published literature, software must be made available to editors and reviewers. We strongly encourage code deposition in a community repository (e.g. GitHub). See the Nature Portfolio guidelines for submitting code & software for further information.

Data

Policy information about availability of data

All manuscripts must include a data availability statement. This statement should provide the following information, where applicable:

- Accession codes, unique identifiers, or web links for publicly available datasets
- A description of any restrictions on data availability
- For clinical datasets or third party data, please ensure that the statement adheres to our policy

All next-generation sequencing data generated as part of this study have been deposited in the Gene Expression Omnibus (GEO) under following accession numbers: GSE287493, GSE286895, GSE287206, GSE286894, GSE286923, GSE287492, GSE287494. Previously published ChIP-seq, ATAC-seq and MNase-seq data used in this study is available under the following GEO accession numbers: GSE167632, GSE168142, GSE168141, GSE201852, and GSE120131. The mass spectrometry proteomics data have been deposited to the ProteomeXchange Consortium via the PRIDE partner repository with the dataset identifier PXD067538. Unique reagents such as OCT4 mutants are available from the authors. All sequencing data were aligned to the mouse reference genome MGSCv37 (mm9) (PRJNA20689) or the human genome assembly GRCh37 (hg19) (PRJNA438682). Source data are provided with this paper.

Research involving human participants, their data, or biological material

Policy information about studies with human participants or human data. See also policy information about sex, gender (identity/presentation), and sexual orientation and race, ethnicity and racism.

Reporting on sex and gender	N/A
Reporting on race, ethnicity, or other socially relevant groupings	N/A
Population characteristics	N/A
Recruitment	N/A
Ethics oversight	N/A

Note that full information on the approval of the study protocol must also be provided in the manuscript.

Field-specific reporting

Please select the one below that is the best fit for your research. If you are not sure, read the appropriate sections before making your selection.

[x] Life sciences [ ] Behavioural & social sciences [ ] Ecological, evolutionary & environmental sciences

For a reference copy of the document with all sections, see nature.com/documents/nr-reporting-summary-flat.pdf

Life sciences study design

All studies must disclose on these points even when the disclosure is negative.

Sample size	No specific statistical calculations were performed to predetermine sample size; however, sample size was determined based on established protocols. Sample sizes are indicated in each Figure legend. Generally, three to six biological replicates were used. This was determined according to established methods in the field and previous experience such as that in (Roberts et al; DOI: 10.1038/s41556-021-00727-5), which allowed us to predetermine the number of sample size of ChIP-seq, ATAC-seq and RNA-seq experiment. For iPSC, ESCs, iTSCs, at least three independent cell lines were generated to confirm the results.
Data exclusions	Data from all replicates were used and no data were excluded. All experiment included positive, negative or internal controls. However, ChIP-seq and ATAC-seq data that overlapped with ENCODE black list were excluded from our analysis.
Replication	All replication attempt were successful. Each Experiment was repeated as indicated in the figure legends and the Methods section.

Randomization	Random DNA sequences were used as controls for motif enrichments and other analysis as indicated in the methods section.
Blinding	For mutation screens, ChIP-seq, RNA-seq, ATAC-seq and MS, blinding was not necessary as there was no group attribution and all analyses were fully automated.<br>For chimera and teratoma analysis, cell line genotypes were unknown to the investigator during the experiments and analyses processes.

Reporting for specific materials, systems and methods

We require information from authors about some types of materials, experimental systems and methods used in many studies. Here, indicate whether each material, system or method listed is relevant to your study. If you are not sure if a list item applies to your research, read the appropriate section before selecting a response.

Materials & experimental systems

n/a	Involved in the study	
☐	☒	Antibodies
☐	☒	Eukaryotic cell lines
☒	☐	Palaeontology and archaeology
☐	☒	Animals and other organisms
☒	☐	Clinical data
☒	☐	Dual use research of concern
☒	☐	Plants

Methods

n/a	Involved in the study	
☐	☒	ChIP-seq
☒	☐	Flow cytometry
☒	☐	MRI-based neuroimaging

Antibodies

Antibodies used	All antibodies with catalogue numbers are listed in Materials and Methods section including: OCT4 antibody (#ab19857; Abcam), anti-SOX2 0.5 µg/ml (#AF2018; R&D systems), anti-KLF4 0.5 µg/ml (#AF3640, R&D systems), anti-MYC 1 µg/ml (#AF3696; R&D systems), anti-Nanog 0.5 µg/ml (#14-5761-80; eBioscience), anti-NANOG (ab109250, Abcam), anti-human GAPDH (GTX627408; GeneTex Inc), anti-human TNIP2 (#15459-1-AP, ProteinTech), anti-human ETV4 (#PA5-79223, ThermoScientific), anti-human XPO6 (#ab72333, Abcam), anti-human UFD1L (#10615-1-AP, ProteinTech), anti-human histone H3 (#ab24834, Abcam) and anti-human lamin (0.5 µg/mL; #ab16048, Abcam).
Validation	All antibodies were obtained from commercial sources and were validated by the company; refer to the company website for detailed validation analysis. The antibodies were also validated in our laboratory by ChIP-seq (specific peaks), western blots (one band corresponding to the expected size), and immuno-fluorescence (nuclear staining).

Eukaryotic cell lines

Policy information about cell lines and Sex and Gender in Research

Cell line source(s)	Human iPSCs were generated in Abdenour Soufi's laboratory at the University of Edinburgh.<br>Human ES MasterShef-7 cell line were established in Prof. Harry Moore's laboratory at the University of Sheffield.<br>Human primary fibroblast cell lines were derived in RBiomedical, Edinburgh, UK, from skin samples from anonymous donors undergoing routine surgery at the Edinburgh Royal Infirmary.<br>Conditionally null Pou5F1 ESC line (ZHBTc4.1) were established by Niwa et al (DOI:10.1038/74199) in Austin Smith laboratory and obtained from Ian Chambers Laboratory at the University of Edinburgh.<br>E14tg2α mES cells were obtained fromI an Chambers Laboratory at the University of Edinburgh.<br>Mouse embryonic fibroblasts (MEFs) were derived from 129 strain mice kept at the University of Edinburgh animal facility.<br>Cas9-TNG-MKOS-MEFs and Cas9-TNG-ESCs were established in the Kaji laboratory at the University of Edinburgh.<br>HEK 293T cell lines were used for lentivirus production. These cells are isolated from human embryonic kidneys (HEK) and the 293T cells are transformed with large T antigen. HEK 293T cell line was originally created in Michele Calos's lab at Stanford (DuBridge et al; doi:10.1128/MCB.7.1.379) and obtained from the Kaji laboratory.<br>Human iTSCs were generated in Yossi Buganim's laboratory in the Hebrew University and Hadassah Medical Center.
Authentication	MEFs were authenticated by the University of Edinburgh Animal facility by genotyping DNA extracted from the tail clip or ear notch using PCR. The HEK 293T cell line was authenticated previously (DuBridge et al; doi:10.1128/MCB.7.1.379).All iPS lines generated from human and mouse fibroblasts and ES lines were authenticated by PCR genotyping and Sanger sequencing for OCT4 mutations and knockouts. Human ES cells were authenticated in Prof. Harry Moore's laboratory at the University of Sheffield. Human iTSCs were authenticated by PCR and immunofluorescence in Yossi Buganim's laboratory as previously reported (Benchetrit et al; DOI: 10.1016/j.stem.2019.03.018).<br>newly generated mouse ESC lines were authenticated by clonal Sanger sequencing and PCR genotyping.
Mycoplasma contamination	All cells were routinely checked for Mycoplasma contamination and tested negative.
Commonly misidentified lines<br>(See ICLAC register)	No misidentified cell lines were used in this study.

Animals and other research organisms

Policy information about studies involving animals; ARRIVE guidelines recommended for reporting animal research, and Sex and Gender in Research

Laboratory animals	Mouse embryonic fibroblasts (MEFs) were derived from 129 mouse strain. CD-1 mouse strain was used to generate Chimaeras.
Wild animals	N/A
Reporting on sex	N/A
Field-collected samples	N/A
Ethics oversight	All animal experiments for the iPSC generation from mouse embryonic fibroblasts and chimera generation were approved by the University of Edinburgh Animal Welfare and Ethical Review Body, performed at the University of Edinburgh, and carried out according to regulations specified by the Home Office and Project License.<br>Teratoma assays were performed in compliance with the joint ethics committee (IACUC) of the Hebrew University and Hadassah Medical Center and the National ethic committee (Israel health ministry) and NIH, which approved the study protocol for animal welfare. The Hebrew University is an AAALAC international accredited institute.<br>All animals were housed and treated in accordance with the veterinary guidelines and regulations of the University of Edinburgh and the Hebrew University.

Note that full information on the approval of the study protocol must also be provided in the manuscript.

Plants

Seed stocks	N/A
Novel plant genotypes	N/A
Authentication	N/A

ChIP-seq

Data deposition

☒ Confirm that both raw and final processed data have been deposited in a public database such as GEO.

☒ Confirm that you have deposited or provided access to graph files (e.g. BED files) for the called peaks.

Data access links<br>*May remain private before publication.*	https://www.ncbi.nlm.nih.gov/geo/query/acc.cgi?acc=GSE287493<br>https://www.ncbi.nlm.nih.gov/geo/query/acc.cgi?acc=GSE286923
Files in database submission	GSM8733403_OCT4-WT_ChIP_OSKM48h.SeqDepthNorm.bw<br>GSM8733404_Input_DNA_OSKM48h.SeqDepthNorm.bw<br>GSM8733405_OCT4-lin29-24_ChIP_OSKM48h.SeqDepthNorm.bw<br>GSM8733406_OCT4-lin95-117_ChIP_OSKM48h.SeqDepthNorm.bw<br>GSM8733407_OCT4-lin-mini_ChIP_OSKM48h.SeqDepthNorm.bw<br>GSM8733408_OCT4_ChIP_hESCs.SeqDepthNorm.bw<br>GSM8733409_Input_DNA_hESCs.SeqDepthNorm.bw<br>GSM8746496_Oct4_mut_mESC_CL1.SeqDepthNorm.bw<br>GSM8746497_Oct4_mut_mESC_CL3.SeqDepthNorm.bw<br>GSM8746498_Oct4_mut_mESC_CL7.SeqDepthNorm.b
Genome browser session<br>(e.g. UCSC)	N/A

Methodology

Replicates	Three ChIP replicates were pooled to make a DNA library for each ChIP-seq experiment and two independent replicates were carried out.
Sequencing depth	Around 50-60 million pair-end reads were obtained on average from each ChIP-seq.

Antibodies	OCT4 antibody (#ab19857; Abcam)
Peak calling parameters	Duplicates were removed from the aligned pair-end BAM files using Picard prior to peak calling. TF peaks (sample files) showing significant enrichment over input DNA (control files) obtained from the same cells were called using MACS2 and a fragment size of 200 bp (--nomodel --extsize 200) and were controlled to q value (minimum FDR) cut-off of 0.01 (-q 0.01). The peaks that overlapped with the ENCODE mm9 or hg19 blacklist were removed.
Data quality	ENCODE data quality standards for Transcription factor ChIP-seq were followed: https://www.encodeproject.org/chip-seq/transcription-factor-encode4/
Software	FASTQC, Bowtie, Bedtools, Picard, MACS, DeepTools, Qualimap, SAMTool2, MEME, R v3.6 (various R packages as indicated in the methods), BEDOPS and Cutadapt.
